# A rare image of zinc-responsive acral hyperkeratosis

**DOI:** 10.11604/pamj.2025.50.27.45731

**Published:** 2025-01-15

**Authors:** Abhishek Patil, Sourabh Deshmukh

**Affiliations:** 1 Department of Kayachikitsa, Mahatma Gandhi Ayurved College Hospital and Research Centre, Salod (H), Wardha, Datta Meghe Institute of Higher Education and Research, Maharashtra, India

**Keywords:** Hepatitis C infection, necrolytic acral erythema, zinc

## Image in medicine

Acral hyperkeratosis that responds to zinc usually manifests as symmetrically distributed, long-lasting, well-defined hyperpigmented plaques over the acral areas of the body. It is a condition that causes thickening of the skin on the hands and feet. Zinc deficiency can be a cause of acral hyperkeratosis. A 50-year-old man came in with a six-month-old, slightly itchy, scaly, raised lesion over both feet which were darkly coloured and somewhat irritating. A cutaneous examination of the dorsum of the feet revealed bilaterally symmetrical, well-demarcated hyperpigmented plaques. The patient was treated using oral zinc with 200mg of zinc sulfate twice daily and 10% urea-containing emollient for local application. He got significant relief from raised lesions and hyperpigmented plaques.

**Figure 1:**
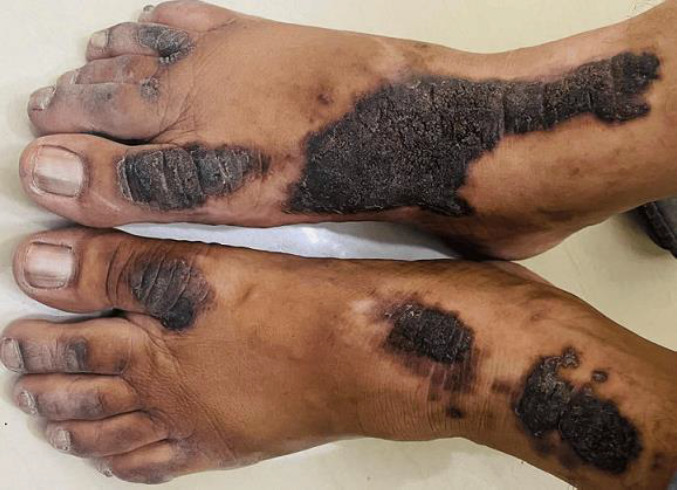
feet with raised lesions and hyperpigmented plaques

